# The challenge of antimicrobial resistance in intensive care setting

**DOI:** 10.2478/jccm-2025-0020

**Published:** 2025-04-30

**Authors:** Prisco Piscitelli, Vincenzo Costigliola, Leonard Azamfirei

**Affiliations:** European Medical Association (EMA), Bruxelles, Belgium; Department of Psychology and Health Sciences, Pegaso University, Naples, Italy; Department of Anesthesiology and Intensive Care, George Emil Palade University of Medicine, Pharmacy, Science and Technology of Targu Mures, Romania

Antimicrobial resistance (AMR) is a growing global health crisis, also in the frame of One Health perspective [1]. This problem represents a dramatic emergency in critical care settings like intensive care units (ICUs), where patients are most vulnerable, also because of persistent lack in the pharmaceutical pipeline for the development of new antibiotics [2,3,4]. ICUs are the front line in managing patients with life-threatening conditions, such as severe infections, trauma, and organ failure. Therefore, ICUs present unique conditions – as patients are critically ill, immunocompromised, and often subjected to invasive procedures and extended hospital stays — provide an ideal environment for the emergence and spread of resistant pathogens. AMR poses significant challenges for healthcare providers in ICUs, where timely and effective antibiotic treatment is crucial, directly impacting on clinical outcomes, mortality rates and increased healthcare costs [5].

## Key Problems in Antimicrobial Resistance in ICUs

The high frequency of patients undergoing invasive procedures in intensive care often requires invasive devices such as ventilators, catheters, and dialysis machines. These devices provide a direct pathway for bacteria to enter the body, increasing the likelihood of infections. Additionally, prolonged use of these devices raises the risk of developing infections caused by resistant pathogens. Moreover, many ICU patients are immunocompromised due to conditions such as cancer, organ transplantation, or critical illness. These patients have a weakened immune system, making it harder for their bodies to fight off infections. As a result, infections caused by resistant microorganisms can quickly escalate and become life-threatening.

One of the most pressing concerns in ICUs is the spread of multidrug-resistant organisms (MDROs), including Methicillin-resistant Staphylococcus aureus (MRSA), Vancomycin-resistant Enterococci (VRE), and Carbapenem-resistant Enterobacteriaceae (CRE). These bacteria are resistant to multiple classes of antibiotics, significantly limiting treatment options. In ICUs, where the use of antibiotics is often intensive and broad-spectrum, the development and spread of these resistant strains are accelerated.

At the same time, ICUs are at risk of inappropriate and overuse of antibiotics, which are often prescribed empirically, meaning they are given based on the assumption that an infection is present, even before its cause is definitively identified. The overuse and misuse of antibiotics can drive the selection of resistant bacteria. Additionally, prolonged or incorrect antibiotic therapy contributes to the development of resistance. Finally, our concern should also include the problem of cross-transmission between patients, as the close proximity of ICU patients, combined with the frequent need for healthcare workers to move between patients, increases the risk of cross-transmission of resistant microorganisms. Inadequate infection control measures, such as poor hand hygiene or improper use of personal protective equipment, can further exacerbate this problem.

## Major Antibiotic-Resistant Pathogens in ICUs

Methicillin-Resistant Staphylococcus aureus (MRSA) is one of the most common causes of healthcare-associated infections in ICUs, leading to conditions like pneumonia, bloodstream infections, and surgical site infections. It is resistant to methicillin and other beta-lactam antibiotics, making it difficult to treat. Carbapenem-Resistant Enterobacteriaceae (CRE), which includes Klebsiella pneumoniae and Escherichia coli, is a major concern due to its resistance to carbapenems, a class of antibiotics considered last-line treatments for many bacterial infections. These pathogens can cause severe infections such as sepsis and pneumonia, particularly in immunocompromised ICU patients. Vancomycin-Resistant Enterococci (VRE) VRE are responsible for a range of infections, including urinary tract infections and bloodstream infections. These bacteria are resistant to vancomycin, a key antibiotic used to treat enterococcal infections, and they can spread rapidly in hospital environments, especially in ICUs. Acinetobacter baumannii is a highly resistant pathogen that can cause pneumonia, bloodstream infections, and wound infections, particularly problematic in ICUs due to its ability to survive in harsh hospital environments and its resistance to multiple antibiotics, including carbapenems and colistin [6,7].

## Possible Solutions to Counteract Antimicrobial Resistance in ICUs

One of the most effective strategies to combat AMR in ICUs is the implementation of antibiotic stewardship programs (ASPs). These programs involve a team of experts who oversee and optimize antibiotic use, ensuring that antibiotics are prescribed only when necessary, and that the right drug, dose, and duration are chosen [8]. ASPs have been shown to reduce the incidence of infections caused by resistant microorganisms and improve patient outcomes. Strict infection control practices, including hand hygiene, the use of personal protective equipment (PPE), and environmental cleaning, are also essential to reduce the spread of resistant bacteria in ICUs.

Isolation precautions for patients with known resistant infections is compulsory to minimize cross-transmission. The fight against AMR can benefit from the use of rapid diagnostic tests which can help to identify the causative organisms of infections quickly, allowing for more targeted antibiotic therapy. This approach reduces the unnecessary use of broad-spectrum antibiotics and helps ensure that patients receive the most appropriate treatment. Regular surveillance of resistant pathogens within the ICU is crucial for early detection and containment. Actually, monitoring antimicrobial resistance patterns allows healthcare providers to adjust their infection control and treatment strategies accordingly [9]. The pipeline for new antibiotics is currently limited, and there is an urgent need for innovative therapies to address resistant infections as the development of new antibiotics and alternative therapies is critical in the fight against AMR.

## Conclusion

Antimicrobial resistance in intensive care units poses a significant threat to patient safety and the effectiveness of healthcare systems worldwide. The emergence of multidrug-resistant organisms and the complex nature of ICU care create a challenging environment for managing infections. However, through a combination of strategic interventions such as antibiotic stewardship, improved infection control practices, rapid diagnostics, and ongoing research, it is possible to mitigate the impact of AMR and improve outcomes for critically ill patients. The fight against antimicrobial resistance requires a concerted, global effort involving healthcare providers, researchers, policymakers, and the public. There is an urgent need for the development of new antibiotics, especially those that can target resistant organisms like MRSA, CRE, and VRE. Additionally, research into alternative therapies, such as bacteriophage therapy, antimicrobial peptides, and vaccines, may offer new ways to combat resistant infections. Ongoing education and training for healthcare workers on the risks of AMR and the importance of proper antibiotic use are essential. Empowering clinicians with the knowledge and tools to make informed decisions can significantly reduce the misuse of antibiotics.
